# Breadth of tuning in taste afferent neurons varies with stimulus strength

**DOI:** 10.1038/ncomms9171

**Published:** 2015-09-16

**Authors:** An Wu, Gennady Dvoryanchikov, Elizabeth Pereira, Nirupa Chaudhari, Stephen D. Roper

**Affiliations:** 1 Graduate Program in Neurosciences, Miller School of Medicine, University of Miami, Miami, Florida 33136, USA; 2 Department of Physiology and Biophysics Miller School of Medicine, University of Miami, Miami, Florida 33136, USA

## Abstract

Gustatory stimuli are detected by taste buds and transmitted to the hindbrain via sensory afferent neurons. Whether each taste quality (sweet, bitter and so on) is encoded by separate neurons (‘labelled lines’) remains controversial. We used mice expressing GCaMP3 in geniculate ganglion sensory neurons to investigate taste-evoked activity. Using confocal calcium imaging, we recorded responses to oral stimulation with prototypic taste stimuli. Up to 69% of neurons respond to multiple tastants. Moreover, neurons tuned to a single taste quality at low concentration become more broadly tuned when stimuli are presented at higher concentration. Responses to sucrose and monosodium glutamate are most related. Although mice prefer dilute NaCl solutions and avoid concentrated NaCl, we found no evidence for two separate populations of sensory neurons that encode this distinction. Altogether, our data suggest that taste is encoded by activity in patterns of peripheral sensory neurons and challenge the notion of strict labelled line coding.

## Abstract (web summary)

How taste information is encoded and transmitted from the periphery to the cortex is not well understood. Here the authors provide evidence for population-based coding of taste by demonstrating that more than half of individual geniculate ganglion neurons are broadly tuned to basic taste stimuli.

## Graphical abstract

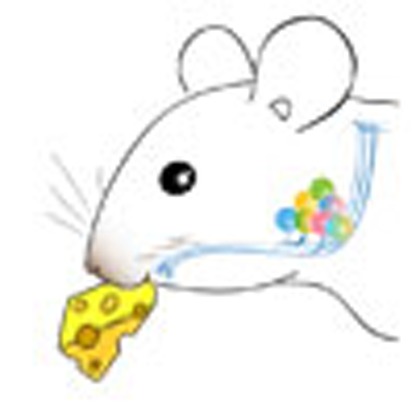

Taste buds are the peripheral end organs of gustation in mammals. They guide an organism to distinguish basic tastes such as sweet, salty, sour, bitter and umami. Approximately 50–100 bipolar epithelial cells cluster tightly together to form a taste bud. When activated by appropriate taste stimuli, excitable cells of the bud signal to nerve fibres that course between the sensory cells^1^. These taste afferents are the peripheral processes of pseudounipolar neurons with cell bodies located in the geniculate or petrosal cranial ganglia, and central projections that terminate in the hindbrain (nucleus of the solitary tract). In mice, each taste bud is innervated by about five neurons^2^, which suggests that each sensory fibre branches and connects to multiple cells. Whether individual afferent neurons connect with and receive signals exclusively from one type (for example, sweet sensing) of taste cell has not been addressed to date.

The logic used by the taste system to code taste quality has been studied at levels from the peripheral receptors to cortical circuits. There is consensus that in taste buds, some receptor cells are mostly, but not absolutely, ‘tuned’ to specific taste qualities. For example, there are taste receptor cells that respond mainly to sucrose, others respond to subsets of bitter compounds and yet others to glutamate and related amino acids (umami)^3^. The relatively narrow tuning and relatively non-overlapping patterns of expression of G protein-coupled taste receptors^4^ makes it attractive to imagine that ‘labelled’ peripheral receptors transmit discrete channels of information along afferent fibres that serve as ‘labelled lines’ (for example, ‘sweet’, ‘bitter’, ‘umami’ and so on) to encode taste^5^. Yet, there is abundant evidence that refutes this view. For example, many taste bud cells express receptors for more than one taste quality^6^,^7^,^8^,^9^ and/or respond to multiple taste stimuli^3^,^6^,^10^,^11^. And one class of taste bud cells (so-called Type III cells) consistently responds to multiple taste stimuli by integrating signals from other taste receptors via paracrine interactions within the taste bud^3^,^12^. The broad tuning of Type III cells is particularly difficult to reconcile with labelled line coding. Beyond taste buds, taste afferents that transmit peripheral signals to the brain appear less narrowly tuned. Single-unit recordings have revealed that many sensory axons respond to two or more different taste qualities^13^,^14^,^15^,^16^,^17^,^18^,^19^,^20^,^21^. Neurons in the hindbrain and cortex have progressively more broad response profiles^22^,^23^,^24^,^25^,^26^, an observation antithetical to labelled line coding. In short, how taste is encoded and transmitted from peripheral receptor cells to the brain remains unresolved; an early stage of this information stream is the focus of this study.

In this report we imaged taste-evoked responses in large assemblies of taste afferent neurons in the mouse geniculate ganglion, which transmit taste signals from the peripheral end organs into the hindbrain. The methodology is based on confocal calcium imaging of neurons expressing GCaMP3 selectively in sensory neurons^27^ and a surgical method to expose the geniculate ganglion in anaesthetized mice. Our data indicate that about half of the gustatory sensory ganglion neurons are tuned to a single-taste quality, the remaining being responsive to multiple tastants. Moreover, these proportions vary with the concentration of taste stimulus applied, possibly explaining why some investigators report ‘labelled line’ characteristics for gustatory sensory afferent coding. Portions of this report have previously appeared in abstract form^28^.

## Results

### Evoked Ca^2+^ signals in geniculate ganglion neurons

**Figure 1:**
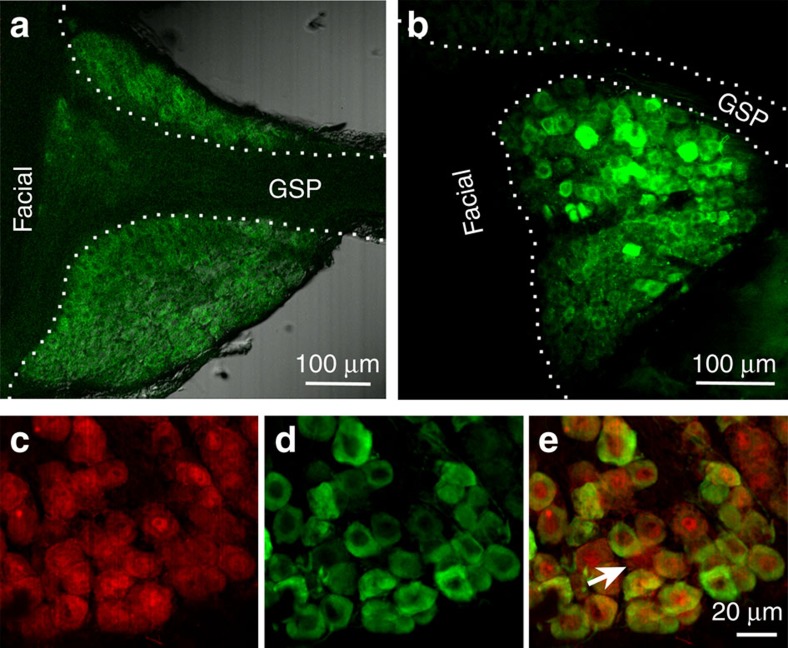
Geniculate ganglia from GCaMP3 mice express Ca^2+^ reporter in nearly every sensory neuron. (**a**) A dissected ganglion viewed with differential interference contrast and fluorescence optics to image GCaMP3. The geniculate ganglion lies at the junction of the facial nerve and the smaller greater superficial petrosal (GSP) nerve. (**b**) A geniculate ganglion imaged *in situ* in a living, anaesthetized GCaMP3 mouse. This is a Z projection (merged stack of several confocal optical sections). Dashed lines show positions of Facial and GSP nerves. (**c**) High magnification of a geniculate ganglion immunostained with anti-NeuN to identify neurons. (**d**) Same section immunostained with anti-GFP to identify cells expressing GCaMP3. (**e**) Merge of **c** and **d**. Arrow in e points to a rare case where a neuron appears not to express GCaMP3. In two representative sections of a geniculate ganglion from a GCaMP3 mouse, coimmunostained with anti-NeuN and anti-GFP as here, 314 of 318 neurons (99%) coexpressed both proteins.

By using transgenic mice that express GCaMP3 in sensory neurons (GCaMP3 mice)^27^, we exposed and imaged taste-evoked activity in large numbers of geniculate ganglion sensory neurons with single-cell resolution and with good temporal detail. GCaMP3 is faithfully expressed in nearly all geniculate ganglion neurons and is readily imaged *in situ* (Fig. 1).

**Figure 2:**
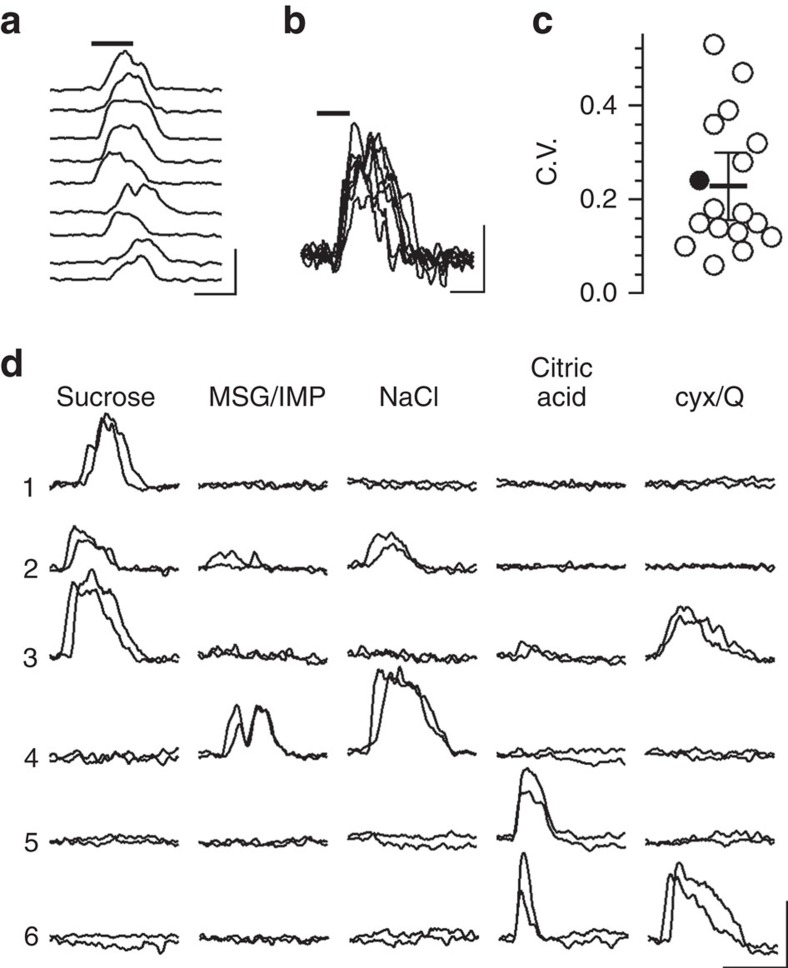
Tastant-evoked responses measured by GCaMP3 fluorescence (Δ*F*/*F*_0_) are robust and reliable. (**a**) Responses elicited by 250 mM NaCl (5 s, perfused into oral cavity at bar shown above traces), recorded simultaneously from nine cells in a geniculate ganglion from one mouse. Calibration, 5 s, 2.0 Δ*F*/*F*_0_. Note that many responses exceed 100% Δ*F*/*F*_0_. (**b**) Responses of a representative geniculate neuron to 7 consecutive trials where 250 mM NaCl was perfused into oral cavity for 5 s (bar). Responses are repeatable (coefficient of variation of peak Δ*F*/*F*_0_=24% for this neuron). Calibration, 5 s, 0.4 Δ*F*/*F*_0_. (**c**) Coefficient of variation (c.v.) for NaCl-evoked responses from experiments similar to those shown in (**b**) (*n*=17 neurons from 2 mice). Bars show mean±95% confidence interval. The filled symbol in **c** is the coefficient of variation from the experiment shown in (**b**). (**d**) Representative examples of GCaMP3 signals recorded from geniculate ganglion neurons in response to prototypic sweet, umami, salty, sour, and bitter taste stimuli. Responses from 6 different neurons from 2 mice are shown. The panel of taste stimuli (top) was presented twice in succession. Neurons 1 and 5 responded only to one taste stimulus (neuron 1, sucrose; neuron 5, citric acid). Neurons 2–4 and 6 responded to multiple taste stimuli. Stimuli were sucrose, 300 mM; MSG, 100 mM (with 1 mM IMP); NaCl, 250 mM; citric acid, 10 mM; cycloheximide (cyx), 1 μM, plus quinine·HCl (Q), 0.3 mM. Calibrations, 10 s, 1.0 Δ*F*/*F*_0_.

Gustatory sensory neurons in the geniculate ganglion innervate taste buds in the palate and anterior tongue. Kim *et al*.^27^ used Ca^2+^ imaging to demonstrate that sensory neurons in the dissected trigeminal ganglion of GCaMP3 mice respond well and with good fidelity to stimulation. Similarly, our first task was to establish the reliability and fidelity of taste-evoked signals in the geniculate ganglion, in our case *in vivo*. Taste stimuli perfused into the oral cavity for epochs lasting 2-, 5- and 10-s evoked large responses in subsets of ganglion cells (Fig. 2). Although repeatable responses were recorded with taste stimulation as brief as 2 s, we concluded that 5-s applications would ensure more reliable distribution of stimuli throughout the oral cavity. Moreover, 5 s closely matches the duration of licking bursts in mice (∼6 s (ref. 29), recognizing that this varies with water deprivation, the tastant solution, and even with the size of the sipping spout^30^). Last, 5-s taste stimulation approximates what others have employed in electrophysiological recordings of geniculate ganglion neurons^31^, thereby facilitating comparisons of our findings with previous studies.

Taste stimulation typically evoked responses in several neurons in the ganglion (Fig. 2a, Supplementary Movie 1). Responses from a given neuron were remarkably consistent when stimulated repeatedly with a single taste compound. A representative neuron displayed a coefficient of variation (c.v.) of 24% in the magnitude of responses (Fig. 2b). The average c.v. for responses from a typical series of recordings was 23% and was not dependent on the taste quality (Fig. 2c). The ability to record reliable and robust responses also did not depend on a neuron’s resting fluorescence (which, parenthetically, reflects a combination of GCaMP3 expression level and resting [Ca^2+^]_i_). At no stage did we observe evoked *decreases* of [Ca^2+^]_i_ in response to taste stimuli.

Next, we tested responses to a panel of five taste stimuli representing prototypic sweet, salty, sour, bitter and umami qualities. To test the stability and reliability of our assay, we presented the sequence of five stimuli twice in succession (Fig. 2d) and calculated the correlation coefficient (*r*^2^) of responses to the first versus the second application across all five stimuli. If each of the five stimuli produced identical first and second responses, then the resulting correlation would be 1. In an initial sample of 163 neurons, the correlation coefficient across the complete panel of stimuli ranged from 0.0018 to 1 (Supplementary Fig. 1). We set a criterion level of *r*^2^>0.20 (mean minus 2 s.d. from the frequency distribution of *r*^2^ values ) for neurons to be accepted and analysed further in this response assay and for all subsequent studies in this report.

**Figure 3:**
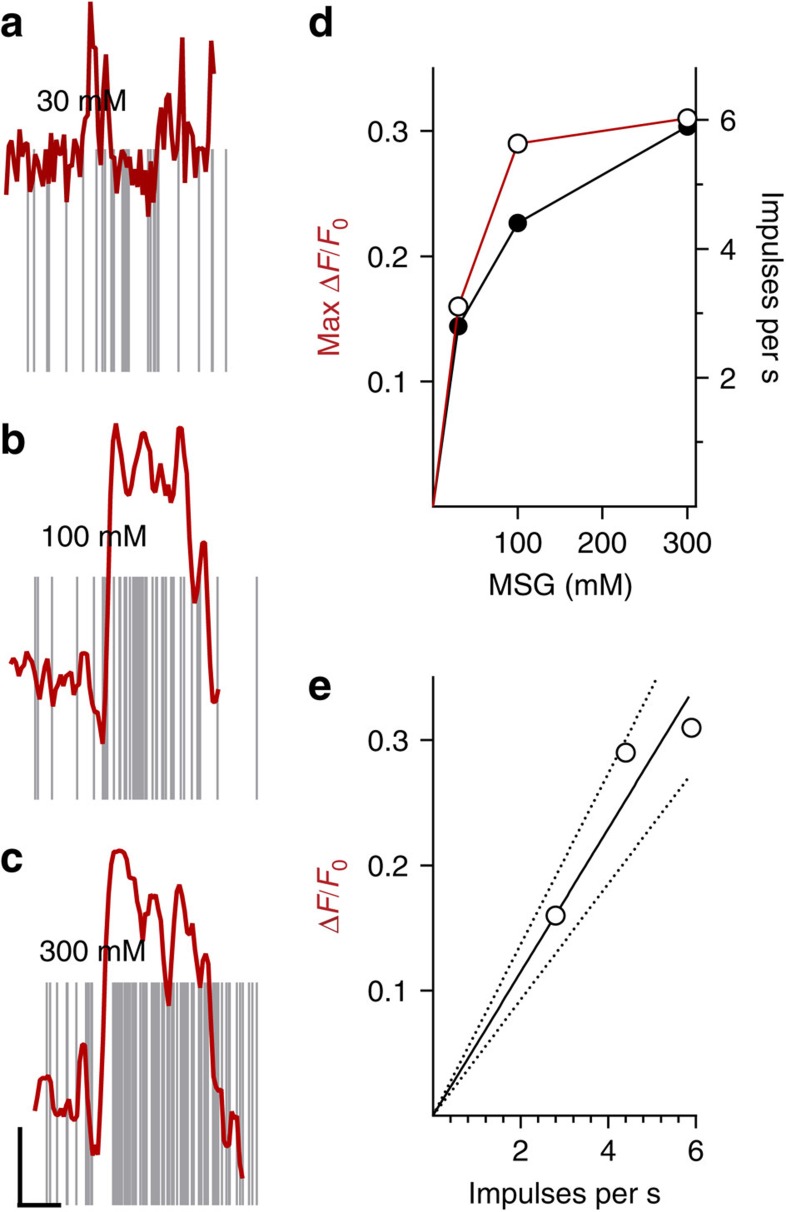
Electrical stimulation of the tongue in a pattern that emulates oral tastant-mediated excitation elicits responses (Δ*F*/*F*_0_) visualized by GCaMP3. (**a**–**c**) Series of electrical pulses (grey lines) applied to the anterior tongue surface, modelled after trains of action potentials recorded from rat geniculate ganglion cells^19^,^20^,^31^. Ganglion cell action potentials were evoked by applying 30, 100 and 300 mM MSG onto a rat tongue in a separate experiment (electrophysiological recordings kindly provided by R. Contreras and A. Nikonov). In response to the trains of electrical pulses to the tongue, the amplitudes of GCaMP3 signals in one representative neuron (Δ*F*/*F*_0_, red traces) increased proportional to the electrical stimulation (pulse frequency, duration). Calibration, 5 s, 0.1 Δ*F*/*F*_0_ (**d**) plot of impulse frequency (closed symbols) and magnitude of GCaMP3 responses (open red symbols) from data in (**a**–**c**). Action potentials were quantified as the average impulses frequency in the initial 5 s of the responses to 30, 100 and 300 mM MSG (**a**–**c**, respectively). (**e**) Correlation of average impulses per second evoked by increasing concentrations of MSG (action potentials in **a**–**c**, closed symbols in **d**) with GCaMP3 responses evoked by electrical stimulation of the tongue (red traces in **a**–**c**, red symbols in **d**). Dashed lines, 95% confidence interval for the linear fit. Note that GCaMP3 responses closely mimic responses recorded electrophysiologically (neuronal action potentials from geniculate neurons).

To validate the technique further, we recorded GCaMP3 fluorescence changes elicited by a pattern of electrical stimulation that was designed to emulate taste stimulation. Specifically, we used a waveform generator to produce brief electrical pulses that were applied to the anterior tip of the mouse tongue with a small-disk electrode. We triggered the waveform generator with actual trains of taste-evoked action potentials previously recorded in separate experiments with microelectrodes from geniculate ganglia (data kindly provided by A. Nikonov and R. Contreras, Florida State University). Our goal was to stimulate taste buds and/or primary sensory afferent fibres with a pattern of electrical pulses that mimicked the actual firing of geniculate neurons during oral taste stimulation. Figure 3a–c shows that GCaMP3 responses increased proportionally to a pattern of electrical pulses that emulated taste stimulation at progressively higher concentrations. There is an excellent correlation between impulses per s and evoked GCaMP3 fluorescence in geniculate neurons (Fig. 3d,e).

**Figure 4:**
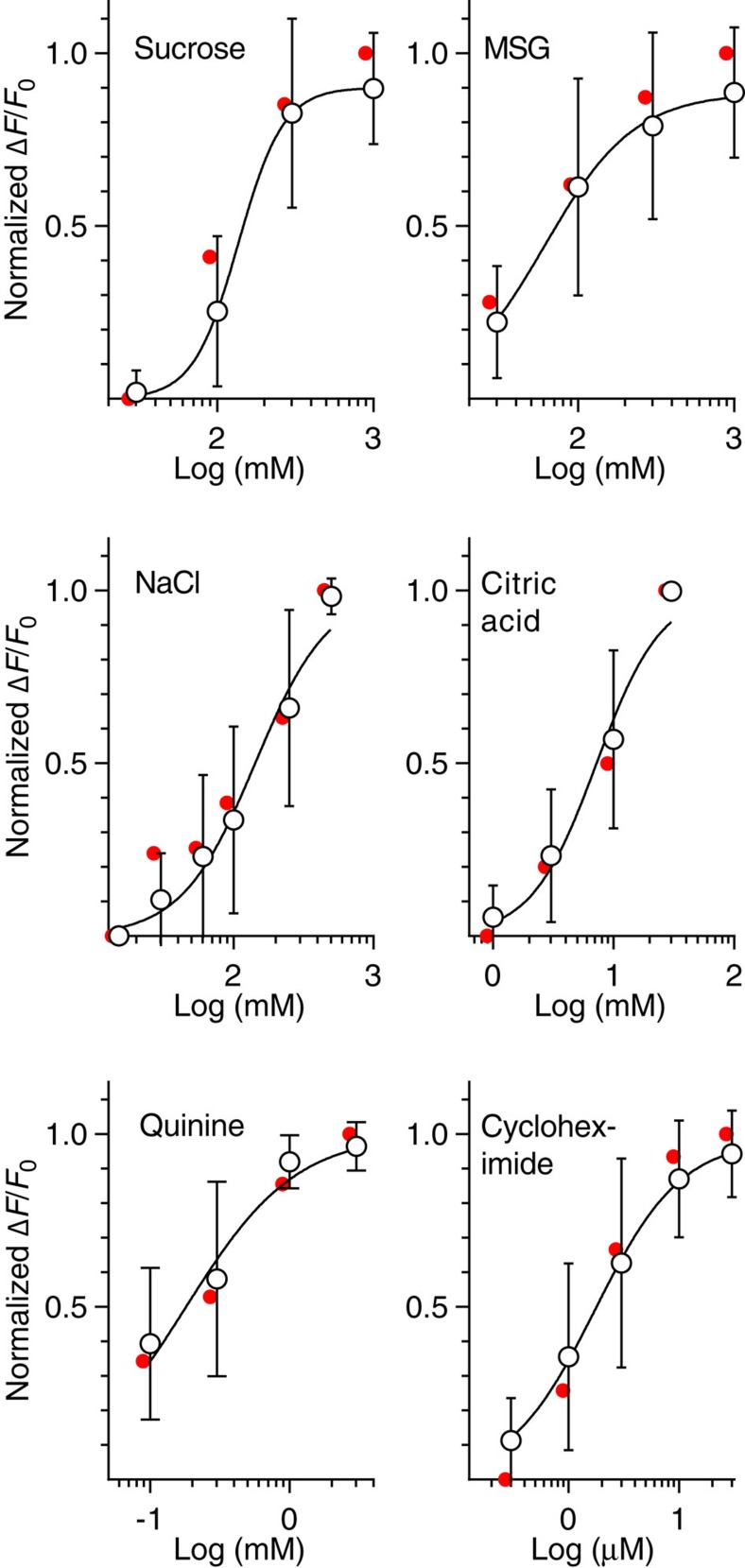
Geniculate ganglion neurons respond to oral tastant stimulation in a concentration-dependent manner. Stimuli were presented at increasing concentrations for each of 6 test compounds representing prototypic sweet, umami, salty, sour, and bitter tastes. All responses (Δ*F*/*F*_0_) from a given neuron were normalized to the peak response for that neuron. To demonstrate the variation in responses, symbols show means±s.d. Red symbols in each panel show responses from one representative individual cell. Lines are best-fit sigmoidal curves with variable slope (*N*=10 to 44 neurons from 3 mice for each curve). EC_50_ values derived from sigmoidal curves are: sucrose, 136 mM; MSG (plus 1 mM IMP), 61 mM; NaCl, 142 mM; citric acid, 7 mM; quinine HCl, 0.2 mM; cycloheximide, 1.7 μM.

A final important benchmark for these studies is that the dynamic range of responses should reflect the range of concentrations to which mice respond behaviourally and also which have been recorded electrophysiologically from gustatory nerves. To this end, we performed concentration–response analyses for five prototypic taste qualities, sweet, salty, sour, bitter and umami (Fig. 4). Importantly, stimulus concentrations for half-maximal responses (EC_50_) corresponded closely in behavioral and electrophysiological studies, and our GCaMP3 imaging (Table 1). This supports the use of functional imaging for detailed quantitative analyses of ganglion cell responses to oral taste stimulation.

### Taste profiles

**Figure 5:**
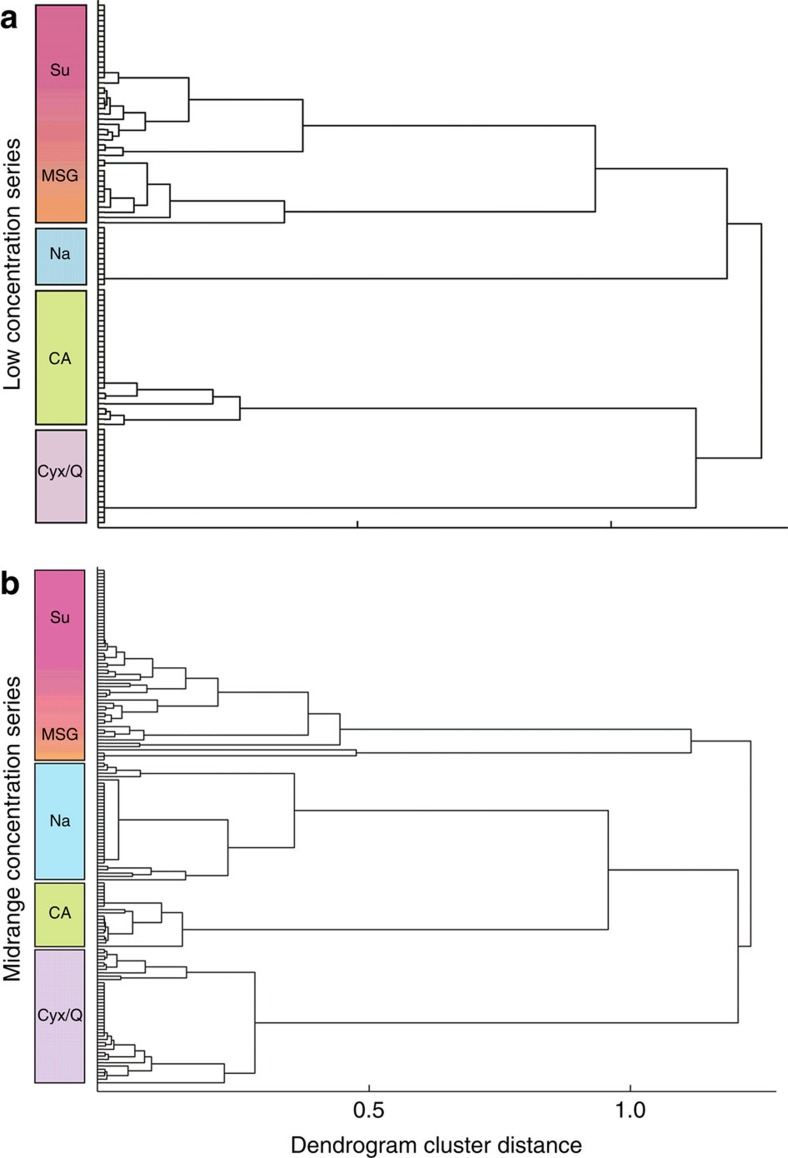
Dendrograms of taste-evoked responses from geniculate ganglion neurons show clustering into sweet, umami, salty, sour and bitter. Five prototypic taste solutions were sequentially perfused over the tongue and palate and responses recorded (see Fig. 2d). Two ranges of stimulus concentrations were compared, (**a**) low (<EC_50_) and (**b**) mid-range (∼1.5 to 2 × EC_50_) as described in the text. Five main clusters are evident: sweet (Su, sucrose); umami (MSG, monosodium glutamate plus IMP); salty (Na, NaCl); sour (CA, citric acid); and bitter (Cyx, cycloheximide; Q, quinine·HCl). Data represent 101 neurons from 12 mice (**a**), and 155 neurons from 9 mice (**b**). Taste stimuli, low concentrations (**a**): 100 mM sucrose; 60 mM NaCl; 3 mM citric acid; mixture of 0.6 μM cycloheximide with 0 or 0.1 mM quinine; 60 mM monosodium glutamate with 1 mM IMP. Taste stimuli, mid-range concentrations (**b**): 300 mM sucrose; 250 mM NaCl; 10 mM citric acid; mixture of 1 μM cycloheximide with 0.3 mM quinine; 100 mM monosodium glutamate with 1 mM IMP.

Having established the reliability and fidelity of measuring taste-evoked GCaMP3 signals in geniculate ganglion cells, we next examined the breadth of tuning for gustatory sensory neurons when the oral cavity was stimulated with different taste compounds. We noticed that some neurons responded to only one prototypic tastant but others had a greater breadth of tuning (Fig. 2d). Thus, we catalogued responses from a sample of geniculate neurons (*N*=101) to each of five prototypic basic tastes—sweet: 100 mM sucrose; salty: 60 mM NaCl; sour: 3 mM citric acid; bitter: mixture of 0.6 μM cycloheximide with 0 or 0.1 mM quinine; and umami, 60 mM monosodium glutamate (MSG) with 1 mM IMP. These concentrations were selected to be slightly below EC_50_ values based on concentration-response curves (Fig. 4). Cluster analysis showed a clear categorization of responses into different classes representing salty, sour, bitter, sweet and umami (Fig. 5a). While sweet and umami form separate clusters, these clusters are closer together than the other taste qualities, as evidenced by the shorter stem separating them.

We further tested whether increasing the concentrations of taste stimuli altered the categorization of responses, for example, by widening the breadth of tuning of ganglion neurons, similar to what is reported for glomeruli in the olfactory bulb with increasing odour intensity presentation^32^. Thus, the study was repeated with another sample of neurons (*N*=155), using mid-range stimulus concentrations, approximately 1.5–2 × EC_50_ values: sweet: 300 mM sucrose; salty: 250 mM NaCl; sour: 10 mM citric acid; bitter: mixture of 1 μM cycloheximide with 0.3 mM quinine; and umami, 100 mM monosodium glutamate with 1 mM IMP. The resultant cluster analysis (Fig. 5b) suggested greater heterogeneity within each category. For example, at low concentrations (Fig. 5a), both NaCl and the bitter mix yield a tight cluster with a single stem. In contrast, at mid-range concentrations (Fig. 5b), each of these stimuli display multiple sub-groupings in the dendrogram. Parenthetically, we note that sour and bitter (aversive) are only slightly more similar to each other than they are too sweet and umami (appetitive). Hence, there is little evidence of strong separation of appetitive from aversive stimuli.

**Figure 6:**
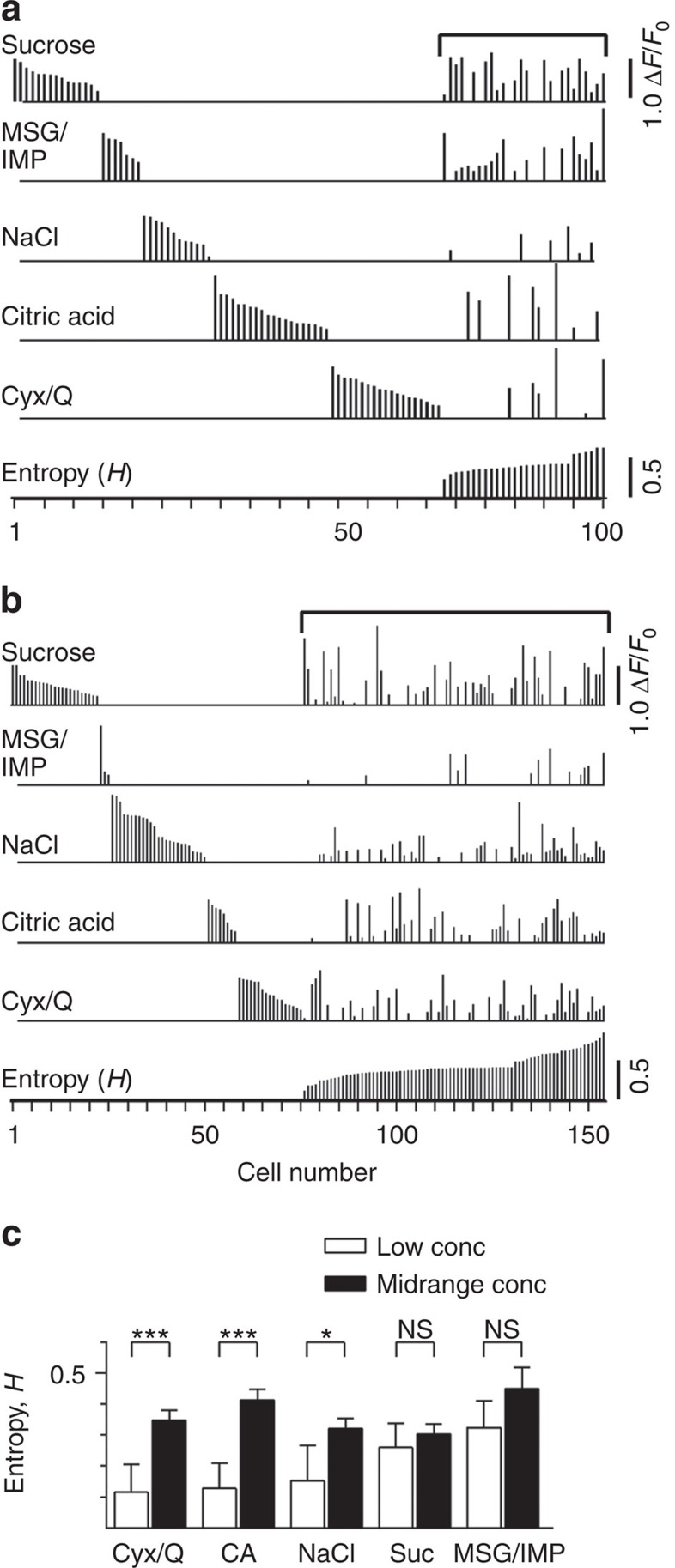
Many neurons in the geniculate ganglia respond to multiple taste stimuli. The same data set as shown in Fig. 5 are represented here in a different manner to reveal the patterns of responses for each individual neuron. Neurons are arranged sequentially from left to right (abscissae). Two different concentrations of taste stimuli were tested: (**a**) low (<EC_50_; *n*=101 neurons) and (**b**) mid-range (∼1.5 to 2 × EC_50_; *n*=155 neurons), as described in the text. For each cell, response amplitude(s) (Δ*F*/*F*_0_) for sucrose, MSG (plus IMP), NaCl, citric acid, and a mixture of cycloheximide and quinine·HCl (Cyx/Q) were plotted on their respective *x* axes aligned above each cell. The bottom line in **a**,**b** plots the entropy (*H*) for each cell. Cells are arrayed from low to high entropy, left to right. Brackets above plots in **a**,**b** indicate cells that respond to multiple taste stimuli (that is, *H*>0). (**c**) mean breadth of tuning (entropy, *H*) for cells exhibiting tastant-evoked responses for the five taste qualities, presented at low versus at mid-range concentrations. Error bars show 95% confidence interval. At the low concentrations (open bars) there were differences among the five tastes (*P*=0.005, Kruskal–Wallis test) with Cyx/Q and CA being the most narrowly tuned categories. At mid-range concentrations (solid bars), there were no significant differences (*P*=0.092, Kruskal–Wallis test). *,*P*<0.05; ***,*P*<0.001; CA, citric acid; Cyx, cycloheximide; MSG, monosodium glutamate; NS, not significant; Q, quinine; Suc, sucrose. Stimulus concentrations as described in text and in Fig. 5.

A way to quantify this heterogeneity or breadth of tuning of geniculate ganglion sensory neurons is to calculate the ‘entropy’, *H*, of neuronal responses to the taste stimuli^33^. *H* varies from 0 to 1. The greater the value of *H*, the broader the tuning; *H*=0 indicates a neuron that is tuned to a single-taste stimulus (that is, a ‘specialist’); *H*=1.0 indicates a neuron that is a complete ‘generalist’, responding equally to all five taste stimuli. Panels a and b in Fig. 6 display the distribution of taste responses for all neurons in the two stimulus concentration ranges, arranged sequentially according to *H* (plotted along the bottom axis). Although there are significant numbers of multiply responding neurons at both stimulus concentrations, the proportion of broadly tuned neurons (*H*>0, brackets at top) was increased nearly twofold at the mid-range stimulus concentrations. Specifically, 28% (28/101) responded to multiple taste stimuli in the lower stimulus concentration series (Fig. 6a) versus 51% (79/155) at mid-range stimulus concentrations (Fig. 6b). These proportions were significantly different (*P*=0.044, Fisher’s exact test). The mean value of *H* in the lower stimulus concentration series was 0.12, while for the mid-range concentration, the mean was 0.23. These means differed significantly (*P*=0.029, two-tailed Student’s *t*-test).

We examined the data set to determine if there was any underlying pattern to the distribution of *H* values among the neurons. Specifically, we analysed whether cells that responded to certain tastants were associated with particular ranges of entropy. When presented with lower concentrations (below EC_50_) of tastants, neurons that responded to cycloheximide/quinine or to citric acid were in general more narrowly tuned (lower *H*) than those responding to other taste qualities, including sucrose (Fig. 6c, open bars). At mid-range stimulus concentrations (Fig. 6c, filled bars), there were no significant differences in the breadth of tuning across neurons responding to different taste qualities.

The above data indicate that a substantial fraction of geniculate gustatory sensory neurons (>1/4 to 1/2) responded to two or more prototypic taste qualities. At mid-range stimulus concentrations, the proportion of broadly responding neurons increases as does the average breadth of tuning for the neurons. For stimulus concentrations 1.5–2 × EC_50_ values, no particular taste was associated with highly tuned (specialist) or broadly responsive (generalist) neurons.

### Altered breadth of tuning in neurons

**Figure 7:**
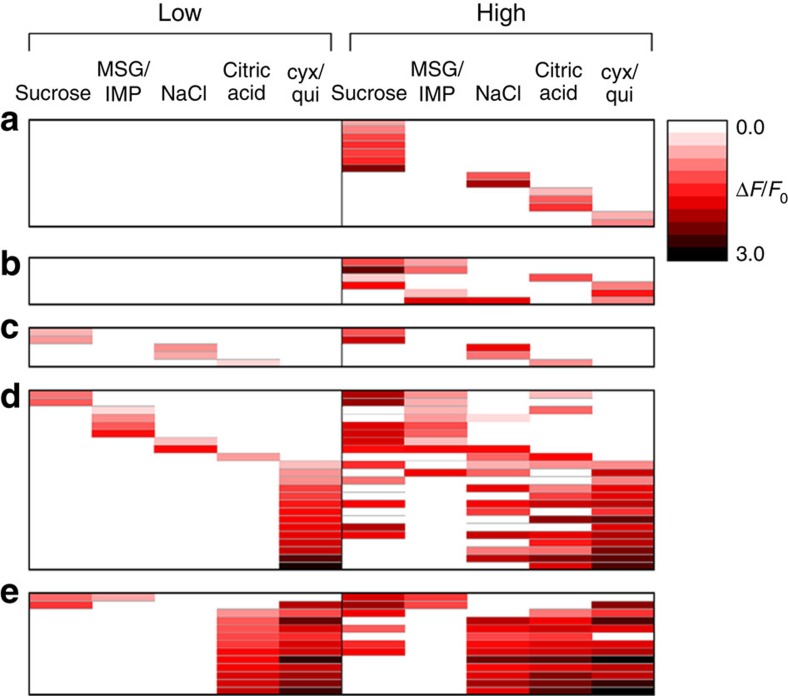
Geniculate neurons become more broadly tuned when the stimulus concentration increases. A panel of five prototypic taste compounds was presented by oral perfusion, as in Figs 4 and 5, twice at a low concentration (near EC_50_) and twice at a concentration near maximum on the concentration-response curves (approximately 3.5–7 × EC_50_). Responses of each neuron were averaged at each concentration and converted into a heat map, with response amplitudes displayed as colour intensities. Columns represent responses to different taste compounds (which are listed across the top). Each row represents the response profile for a single neuron, for a total of 61 neurons. (**a**,**b**) neurons responding only at high stimulus concentrations. These neurons were tuned to a single-taste compound (**a**) or were more broadly responsive (**b**). (**c**–**e**) neurons responding to both low and high concentration stimuli. Some neurons (**c**) were tuned to the same taste compound at low and high concentrations; other neurons (**d**) converted from narrowly tuned to broadly responsive; whereas some neurons (**e**) converted from dual to multiply responsive. A single neuron (top row in **e**) showed dual responses to sucrose and MSP/IMP at both concentrations. Taste stimuli were low: sucrose, 100 mM; MSG/IMP, 60 mM MSG with 1 mM IMP; NaCl, 60 mM; citric acid, 3 mM; cyx/qui; cycloheximide, 0.6 μM, quinine 0.1 mM; high: sucrose, 500 mM; MSG/IMP, 300 mM MSG with 1 mM IMP; NaCl, 500 mM; citric acid, 50 mM; cyx/qui; cycloheximide, 30 μM, quinine 5 mM.

As a final, critical test of taste coding, we studied the tuning properties of individual neurons when presented consecutively with low and high concentrations of taste stimuli. Using our concentration–response data (Fig. 4) for reference, we identified a concentration near or below EC_50_, as before, and another near maximum efficacy (approximately 3.5–7 × EC_50_), but still within concentrations reported for mouse behavioural assays, for each of five stimuli representing different taste qualities. Labelled line coding would predict that each neuron would respond only to one class of stimulus regardless of concentration. However, most geniculate ganglion neurons responded differently when stimulated with low versus high concentrations of the same panel of taste compounds (Fig. 7). Out of a sample of 61 neurons, 41 responded to both low and high stimulus concentrations (Fig. 7c–e). Of these 41 neurons, only 6 responded exclusively to the same stimulus at both concentrations (five ‘specialists’, Fig. 7c and one sucrose–MSG/IMP–responsive neuron, Fig. 7e, first row). Importantly, with increasing stimulus concentration, the majority (35/41, 85%) converted from a specialist to a multiply responsive neuron or from a dual-responding to a broadly tuned neuron. Importantly, while many neurons retained the same ‘best stimulus’ at low and high concentrations, 10/61 neurons switched to a different or multiple ‘best stimuli’. In summary of 61 neurons, 42 (69%) responded to stimuli of two or more qualities. Because perceived taste is concentration invariant for stimuli other than salts (for example, sucrose is sweet, whether at low or high concentrations^34^), the data illustrated in Fig. 7 are compatible with pattern coding^35^ rather than labelled line coding.

### Salt taste

**Figure 8:**
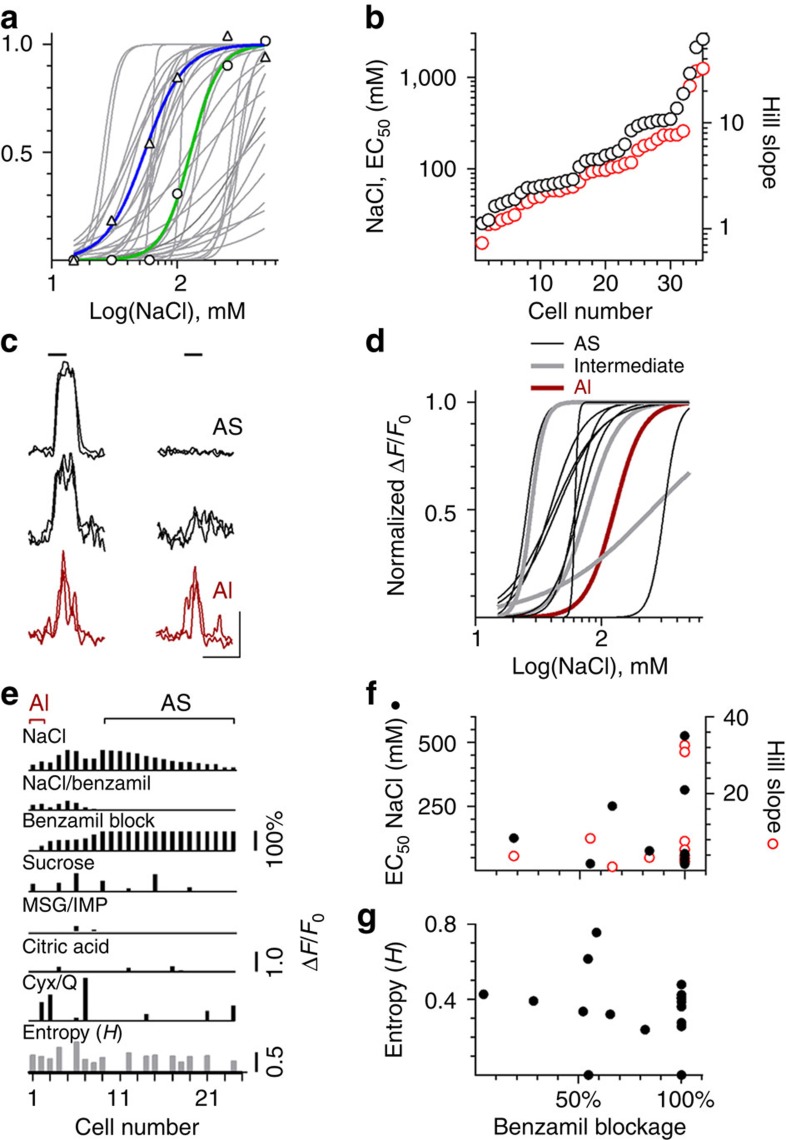
NaCl-evoked responses in geniculate ganglion neurons do not form distinct groups based on concentration-response relations or amiloride-sensitivity. (**a**) NaCl concentration-response relations indicate a wide range of salt sensitivities (*N*=35 neurons from 6 mice). Plots shown in grey are best-fit sigmoidal curves as described in Fig. 5. Two curves (blue, green) are highlighted to show typical data spread for individual cells. (**b**) EC_50_ (black symbols) and Hill slopes (red symbols) obtained from curves in **a**. There is no apparent grouping of NaCl sensitivities. (**c**) NaCl-evoked responses vary in their sensitivity to benzamil (a high-potency amiloride derivate). Bars above traces indicate oral stimulation with 250 mM NaCl. Responses from some neurons are blocked by benzamil (1 μM, top, AS, ‘amiloride-sensitive’), others are resistant (bottom, AI, ‘amiloride-insensitive’) and others are only partially blocked (middle). Replicate responses from one representative cell of each type are shown. Calibration, 10 s, 0.4 Δ*F*/*F*_0_. (**d**) Concentration-response relations for neurons that were also tested for sensitivity to benzamil (1 μM). Thin black lines indicate responses that were blocked by benzamil (AS), thick grey lines show cells with intermediate sensitivity to benzamil, thick red line is a cell that was insensitive to benzamil (AI). Data are from 12 neurons, 3 mice. (**e**) Breadth of tuning for neurons that were also tested for benzamil sensitivity. Neurons are arranged in order of increasing sensitivity to benzamil (top 3 lines), that is, % inhibition of responses evoked by 250 mM NaCl without and with 1 μM benzamil added to stimulus solution. There is no apparent segregation of types of salt-sensitive geniculate ganglion neurons (for example, bitter- or sour-responding or breadth of tuning) based on their sensitivity to benzamil. (**f**) There is no correlation between benzamil sensitivity (*x* axis, % benzamil block of responses to oral stimulation with 250 mM NaCl) and the neuron’s sensitivity to NaCl stimulation (EC_50_, black symbols; *r*=−0.154, Spearman correlation) or with Hill slopes (red symbols; *r*=0.175, Spearman correlation). *N*=13 neurons. (**g**) There is also no correlation between benzamil sensitivity and breadth of tuning (entropy, *H*) of a neuron (*r*=−0.330, Spearman correlation; *n*=24 neurons).

Rodents find dilute solutions of NaCl appetitive and consume them readily, while hypertonic solutions of NaCl are avoided^26^. Accordingly, Oka *et al*.^36^ have predicted that there are two neural pathways for salt sensing: a dedicated labelled line pathway is used for the preferred low concentrations of NaCl. In contrast, aversive, high concentrations of NaCl are carried on lines labelled for bitter and sour tastes. Thus, we tested whether two distinct populations of salt-sensing neurons could be detected. We first examined NaCl concentration–response relations from 35 neurons that met a high criterion of consistency in replicate responses (see Methods, data analysis). These neurons displayed a wide range of NaCl taste sensitivities (Fig. 8a). There was no evidence of grouping into two distinct populations based on their concentration–response curves. EC_50_ values from the curves showed a continuous distribution from 26 mM to >1 M. Similarly, Hill slopes were distributed continuously without forming two populations (Fig. 8b).

In a separate set of neurons, we tested another prediction^36^ that pathways for dilute NaCl are preferentially sensitive to amiloride. We recorded NaCl concentration–response relations and then assessed the effect of benzamil, a high-potency amiloride derivative, on responses to 250 mM NaCl. Benzamil (1 μM) exerted a range of inhibition on NaCl-evoked responses, varying from complete inhibition to no effect (Fig. 8c). Importantly, when we plotted the EC_50_ value for each NaCl-responsive neuron versus the ability of benzamil to inhibit the response of that neuron, there was no correlation between salt sensitivity and benzamil inhibition (Fig. 8d,f; *r*=−0.154, *P*=0.236, nonparametric Spearman correlation, two-tailed, *n*=13).

Finally, we also tested the hypothesis that neurons that respond to concentrated NaCl and are amiloride-insensitive also respond to bitter/acid taste stimulation. Thus, in another experimental series we applied the panel of five prototypic taste stimuli as well as tested the effects of benzamil on NaCl-evoked responses. We queried whether salt-sensitive neurons that responded to multiple taste qualities (high entropy, greater breadth of tuning) were selectively insensitive to benzamil. When the profiles of NaCl-sensitive neurons are organized according to increasing benzamil block (Fig. 8e), or when entropy (*H*) is plotted versus benzamil sensitivity (Fig. 8g), there is no correlation between breadth of tuning and the effects of benzamil. We also specifically tested whether there was any correlation between amiloride (benzamil)-insensitive NaCl responses and sour or bitter taste responses in the same cell. Salt-responsive neurons that also were stimulated by bitter or by sour (citric acid) tastants showed no correlation with benzamil sensitivity (correlation for benzamil sensitivity versus acid responses, *r*=0.067, *P*=0.756; versus bitter responses, *r*=−0.359, *P*=0.085; nonparametric Spearman correlation, two-tailed, *n*=24). In this set of 24 neurons, a subset (*n*=8) responded only to NaCl (not to the other four tastants); all 8 showed nearly complete block of NaCl responses by benzamil (94.4±5.6% block), consistent with previous findings^37^,^38^.

## Discussion

This study introduces *in vivo* Ca^2+^ imaging of large assemblies of gustatory sensory afferent neurons to test fundamental parameters of how taste information is coded from the periphery to the first relay in the brain. We demonstrate geniculate sensory neurons with robust and repeated fluorescent signals in response to oral stimulation with prototypic sweet, salty, sour, bitter and umami taste compounds. The methodology confirms and significantly extends previous findings based on electrophysiological recordings from single afferent axons innervating the tongue^13^,^14^,^15^,^16^,^17^, and from their parent neurons in the geniculate ganglion^18^,^19^,^20^,^21^. Specifically, those studies show there is a range of gustatory ‘tuning’ for geniculate ganglion neurons in rats and mice. There are neurons that are tuned to a single-taste quality, ‘specialists’, and other neurons that respond to two or more taste qualities, ‘generalists’. Our findings, obtained from simultaneous, rather than sequential recordings, clearly show that gustatory tuning of neurons depends on the intensity (concentration) of taste stimulation. With mild stimulation (<EC_50_ concentrations of taste compounds), we observed that 72% of neurons appeared tuned to a single-taste quality. Yet on stronger stimulation (mid-range concentrations, ∼1.5 × 2 × EC_50_), only 49% remained tuned, 51% responded to multiple tastants. Most important, neurons that were highly tuned when presented with low concentrations of taste stimuli converted into more broadly responsive neurons when stimulus concentrations were increased. Response profiles for the vast majority (85%) of neurons that responded to low and high concentrations of stimuli were not stable or hard wired, supporting pattern coding^35^. Collectively, our findings are consistent with some form of combinatorial peripheral sensory coding to discriminate taste qualities (sweet, salty, bitter and so on), in contrast to dedicated labelled line coding. Indeed, in our study, neurons that appeared to be specialists at low stimulus concentration converted into generalists when tested at high concentrations of the same stimuli. This pattern of taste neuron activation is reminiscent of how olfactory neurons, broaden their tuning profiles as odour concentration increases, consistent with a combinatorial coding logic^39^. In contrast, the tuning of vomeronasal neurons is unaltered across pheromone concentrations in a sensory system that displays a more ‘labelled line’ coding system^40^.

The view that peripheral gustatory signalling is encoded by a form of pattern coding was introduced by Pfaffmann^41^ who recorded single fibre taste responses in the cat chorda tympani. Because he observed individual units that responded to combinations of prototypic salty, sour and bitter-tasting compounds, Pfaffmann (*ibid*.) concluded coding ‘does not depend simply on the ‘all or nothing’ activation of some particular fibre group alone but on the pattern of other fibres active.’ Importantly, a single fibre on its own could not accurately and unambiguously encode a given taste quality. Subsequent to these early recordings, Erickson^42^,^43^ offered a specific interpretation of how taste is encoded in the periphery. He introduced ‘across-fibre coding’, a form of pattern coding where ensembles of individual peripheral afferent fibres are required to signal taste quality; no single fibre carries sufficient information to impart taste quality coding. This model is not unlike how colour is encoded with assemblies of photoreceptors and ganglion cells conveying information on hue.

Pattern coding in taste is supported by studies of the central pathways for taste. As peripheral gustatory signals enter the central nervous system and ascend to higher centers from hindbrain to thalamus to cortex, electrophysiological recordings and functional imaging have shown, with rare exception, that central neurons respond to multiple taste qualities, even more broadly so than do peripheral afferent fibres^22^,^23^,^24^,^25^,^26^,^44^. These findings emphasize pattern coding for taste, possibly also involving specific sequences of impulse activity, that is, temporal coding^45^,^46^,^47^.

In contrast to pattern coding is the notion of labelled line coding in taste. Although peripheral gustatory afferent fibres often respond to multiple taste stimuli, many single units were reported to exhibit strongest responses to one taste quality, for example, ‘sweet-best’ or ‘salt-best’ fibres^14^,^48^. Over time, this labelled line aspect was emphasized, particularly for sweet taste^49^, culminating in some authors extrapolating to a strict labelled line interpretation for gustatory coding throughout the nervous system. Taste bud cells with their relatively non-overlapping pattern of taste receptor expression were said to transmit that ‘label’ to precisely dedicated peripheral sensory afferent fibres, and these fibres relay their information along central pathways to cortical neurons that are tightly segregated by these labels^5^,^50^,^51^. In essence, labelled line coding states that there are separate neural pathways dedicated for each taste sub-modality, for example, two for salty, one each for sweet, sour, bitter and umami. In contrast, combinatorial, temporal and other pattern-coding models can incorporate both narrowly and broadly tuned units as are found in other mammalian sensory systems.

Serious challenges for the notion of labelled line coding in taste, include (a) the findings that some taste cells express G protein-coupled receptors for multiple tastes^7^,^8^,^9^, (b) responses of many individual taste bud cells are not precisely tuned to taste stimulation^3^,^10^,^11^,^16^ and (c) decades of electrophysiological and imaging studies indicating that at all levels along the taste axis, individual units respond to multiple taste qualities (see above). The preponderance of evidence supports some form of pattern and/or temporal coding^52^. Our observation of increased breadth of tuning at higher stimulus concentrations and the conversion of specialist neurons to generalists could be construed by some as evidence of non-specific neuronal activation and not informative for taste identification. However, behavioural experiments consistently show that discrimination between different taste qualities improves at higher concentrations^53^,^54^. Thus, the increased entropy in taste neurons at higher stimulus concentration is apparently intrinsic to the logic of taste coding.

During gustatory stimulation in the present experiments, fluorescence changes in geniculate neurons were proportional to impulse activity in these neurons (Fig. 3), further validating the use of GCaMP3 imaging to measure tastant-evoked activity. Yet, the Ca-mediated increases in GCaMP3 fluorescence did not resolve patterns of action potentials. Thus, our findings do not address the contribution of impulse patterning in taste coding. The timing of impulses and temporal patterning appears to be critical in higher brain centers for taste coding^45^,^46^,^47^. However, there is limited evidence that impulse patterning is as critical in the periphery. In human subjects, stimulating a single-taste bud with brief electrical pulses could evoke a sensation of ‘sweet’ that was unchanged when the stimulus rate was altered^55^. That is, the taste quality code remained constant regardless of impulse frequency. Changing the stimulation pattern *per se* was not tested in those experiments. However, the fact that data from calcium imaging closely mirrors that from electrophysiological recordings in geniculate neurons (Fig. 3) suggests that temporal coding in the periphery may not be as important as in higher brain centers.

A recent publication that also used Ca^2+^ imaging of taste-evoked responses in geniculate ganglion cells reached a very different conclusion from ours^5^. Using stimulus concentrations equal to or even higher than our mid-range concentrations, those authors reported that the vast majority of geniculate ganglion neurons were tuned to a single-taste quality (sweet, salty, bitter, sour or umami); only few (27%) responded to two or more tastants. This led the group to interpret their results as evidence for labelled line coding of taste information. The results we present here instead reveal that a significantly greater proportion of gustatory sensory geniculate ganglion neurons respond to multiple tastants (51%, difference between these two percentages is significant at *P*<<0.001), consistent with what electrophysiological recordings have reported in the past (see above). The basis for the difference in findings is not readily apparent. We used similar prototypic taste stimuli at similar, lower, and higher concentrations as did Barretto *et al*.^5^. Moreover, we showed that the incidence of multiple-sensing geniculate neurons *increases* at the higher stimulus concentrations. Different mouse strains are known to vary in their sensitivities for specific taste stimuli^56^, and this may account for differences in results between us and Barretto *et al*.^5^. The mice in our experiments are on a C57BL/6 background, a strain well-characterized for taste thresholds and sensitivities^56^,^57^. The background strain(s) used by Barretto *et al*.^5^ was not specified. Other possible explanations for the difference include that the taste stimuli in Barretto *et al*. (*ibid.*) may not have reached effective concentrations throughout the tongue and palate, resulting in an over-representation of narrowly tuned neurons (Figs 6 and 7). In addition, the stimulus duration (5 versus 2 s), flow rate (3 versus 5–12.5 ml min^−1^), and delivery method (perfusion via oesophageal cannula versus a feeding tube) differed. Apart from the significantly different incidence of broadly tuned geniculate ganglion cells, and thus a major difference in how taste information coding is interpreted, other details of the two reports are similar, such as that there does not appear to be any topographical mapping of taste quality onto the ganglion.

Our observation regarding the similarity of sweet and umami responses in geniculate ganglion neurons (Fig. 5) is fully consistent with prior molecular, physiological, and behavioural evidence. First, T1R1 and T1R2, components of certain umami and sweet receptors, respectively, are coexpressed in many taste cells^6^,^7^,^8^,^9^. Further, functional responses to both sweet and umami stimuli have been recorded from some taste cells^6^, similar to our findings (Fig. 6). In aggregate, these findings suggest a basis for the long-standing observation that in behavioural assays, rodents may generalize or confuse sweet and umami taste qualities^58^,^59^.

Responses to NaCl, corresponding to so-called salt taste, were complex. Concentration–response curves were heterogeneous, similar to what is reported in the gustatory cortex of rodents^60^,^61^. We also confirmed that in our recordings, neurons that responded exclusively to NaCl, in spite of exhibiting diverse EC_50_ values, were highly sensitive to benzamil, consistent with previous characterization of ‘Na-specialist’ or ‘N’ cells^19^,^20^,^37^,^38^. Behavioural data suggest that the amiloride-sensitive Na-sensitive neurons that we and many others have reported are primarily responsible for coding the perceptual quality of NaCl ‘salty’ taste^53^,^54^. While our findings are consistent with the amiloride-sensitivity of NaCl-selective neurons, we found no evidence to support the recent prediction that there are separate streams of information for low- and high-concentration salt taste sensing^36^,^62^. Instead, geniculate ganglion cells manifest a wide range of NaCl concentration–response relations. Moreover, contrary to the inference that only low-salt sensing neurons are amiloride sensitive^36^,^62^, we found no correlation between the amiloride (benzamil)-sensitivity of individual neurons and their respective EC_50_ for NaCl.

We interpret our findings to indicate that there is not a one-to-one link between taste receptor cells and the final output from the taste bud. As a population, taste bud cells are somewhat selectively tuned: Type II taste bud cells are mostly tuned to a single-taste quality, whereas Type III cells, which are fewer in number, respond to many tastes^3^. This is because Type III cells, although intrinsically sensitive to acid stimuli^12^,^63^ also receive ATP-mediated synaptic excitation from surrounding Type II taste cells^3^,^64^. Sensory afferent fibres within the taste bud do not form ultrastructurally identifiable synapses with Receptor cells. Instead, sensory afferent terminals in the taste bud pass close by taste cells and respond to excitatory neurotransmitter(s) secreted through membrane channels. Thus, a single afferent fibre and its parent geniculate ganglion cell might receive *en passant* excitation from multiple taste cells and thereby respond to two or more different taste stimuli^65^. Sensory axons that synapse with presynaptic (Type III) cells would respond to many stimuli. Indeed, Breza *et al*.^31^ speculated that ‘generalist’ (that is, broadly tuned) geniculate ganglion neurons they recorded with microelectrodes were synaptically coupled to Type III (Presynaptic cells). In short, there are potentially several mechanisms in taste buds and in their neural connections that lead to broadly tuned responses in sensory afferent neurons.

In summary, the broad responsiveness of many geniculate ganglion cells to tastants and more importantly, the conversion of narrow to broad tuning with increasing stimulus intensity argues against strict labelled line coding in the taste periphery and points to a more complex mechanism for encoding taste quality.

## Methods

### Animals

Adult transgenic mice (ages 10 weeks to 1 year) of both sexes that express GCaMP3 in sensory neurons were used for this study^27^. All procedures for surgery and euthanasia were reviewed and approved by the University of Miami IACUC committee.

### Surgery

Mice were anaesthetized with ketamine and xylazine (intraperitonially 0.12 mg g^−1^ ketamine, 0.01 mg g^−1^ xylazine) and placed supine on a pad warmed by circulating water. The animal’s core temperature was monitored with a rectal probe and the mouse was maintained between 36.5 and 37.5 ^o^C. We monitored the level of anaesthesia by hind paw withdrawal reflex. A deep surgical plane of anaesthesia was maintained throughout the surgery and recording session by injections of ketamine (0.12 mg g^−1^ ketamine per h, or as required).

The trachea was exposed and cannulated for respiration. The geniculate ganglion was reached by retracting muscles to expose the middle ear on one side, piercing the bulla, and removing a small piece of the thin temporal bone on the opposite wall^18^. As described by Sollars and Hill^18^, a small flexible tube was introduced into the esophagus and passed forward into the oral cavity to deliver taste stimuli uniformly to the palate and tongue, regions innervated by the geniculate ganglion cells. By attaching a small piece of flexible plastic mesh (4 × 7 mM) to the end of the delivery tube in the oral cavity, we optimized the even distribution of taste solutions across the palate and tongue. We monitored access of taste stimuli in preliminary surgeries by observing the spread of a dilute solution of methylene blue over the tongue and palate.

### Functional imaging

The surgically prepared mouse was carefully transferred to the stage of an Olympus FV1000 confocal microscope equipped with a × 20-long working distance objective that allowed us to image most of the exposed ganglion. Confocal scans of GCaMP3-labelled ganglion neurons using 488-nm laser excitation with a 505–605 nm emitter filter were taken at ∼3 hz while taste stimuli were introduced into the oral cavity. Scans were digitized and stabilized using ImageJ 2.0. Fluorescence intensity of regions of interest (ROIs) drawn over individual ganglion neurons were quantified using imageJ and analysed with MatLab software. We used custom Matlab software code for analysing ROI data (see Supplementary Methods). Responses were quantified as peak stimulus-evoked fluorescence change divided by baseline fluorescence (that is, Δ*F*/*F*_0_). Criteria for analysing responses included that Δ*F*/*F*_0_ exceeded three times the s.d. of the baseline and occurred at a consistent latency after stimulus onset. Further, two replicates were obtained for every stimulus. An alternative approach, measuring the area under the curve during the response yielded similar results but was more time consuming and difficult to automate. Latency of responses reflects the interval for stimuli to traverse the deadspace in the perfusion system, including the oesophageal tube.

### Data analysis

To obtain concentration–response relations for taste-evoked signals in geniculate ganglion cells, a given stimulus was presented in an ascending series of concentrations. This was followed by a second replication of the same presentations. Data points for the two replications were averaged. EC_50_ values were obtained from concentration–response relations fit with a variable slope model (Graphpad Prism v.6.02) that derives a Hill Slope from the data, making no *a priori* assumptions of its value. Moreover, for the detailed analyses of NaCl concentration–response relations (Fig. 8), EC_50_ values were only derived from neurons where there was a good correlation in the responses between the two sequential replications of NaCl concentrations (*r*^2^>0.5) and the goodness-of-fit (*r*^2^) for the sigmoidal plot was >0.6.

Entropy (*H*) for a neuron was calculated as follows^33^




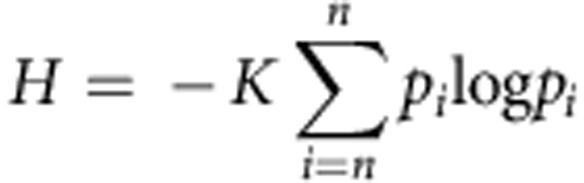




where *n*=number of taste qualities and K is a scaling constant to limit 0>*H*≤1 (*K*=1.43 for *n*=5). *p*_i_ is the proportion of a neuron’s response (Δ*F*/*F*_0_) to the *i*th taste solution relative to the sum across all *n* responses for that neuron. In all cases, we took the mean of two responses (that is, from two separate trials) to calculate *p*_*i*_. *H*=0 indicates that the neuron responded only to one taste quality (highly tuned); *H*=1 indicates the neuron responded equally to all the taste stimuli (broadly tuned).

### Taste stimuli

Stimuli were applied at room temperature for 5 s, preceded and followed by a 55 s rinse with artificial saline. Artificial saline consisted of: NaCl (14.8 mM); KCl (22.1 mM); CaCl_2_ (3.1 mM); MgCl_2_ (0.6 mM). Chemical stimuli for the basic tastes consisted of: sweet–sucrose, 30 mM to 1 M; salty–NaCl, 30 to 500 mM; sour–citric acid, 1 to 30 mM; bitter–a mixture of cycloheximide, 0.3 to 30 μM and quinine·HCl, 0.1 to 3 mM; umami–MSG, 30 to 1 M with 1 mM IMP. Chemical stimuli were delivered by gravity perfusion through a computer-controlled manifold at 3 ml min^−1^. Responses to MSG/IMP were distinguished from responses to NaCl and assigned to the category ‘MSG/IMP’ if the glutamate mixture elicited Δ*F*/*F*_0_>1.5 × that of equimolar NaCl. When 100 mM MSG and 250 mM NaCl were used as stimuli, responses were categorized as ‘MSG/IMP’ if Δ*F*/*F*_0_>1 × that evoked by NaCl.

### Electrical stimulation

To emulate taste-evoked responses in geniculate neurons using electrical stimuli, we applied 10 ms square wave pulses from a stimulator (Grass S9) via a 4 mm silver-silver chloride disk (A-M Systems) applied to the anterior tongue. The stimulus strength was adjusted to just below that which elicited visible muscle contractions. The stimulator was triggered by transistor-transistor logic (TTL) pulses that mirrored action potentials obtained from microelectrode recordings from single geniculate neurons in the rat^19^,^20^. That is, neuronal responses to oral stimultion with 30, 100 and 300 mM monosodium glutamate, applied for 5 s, were digitized and converted into TTL pulses. The microelectrode recordings were kindly provided by A. Nikonov and R. Contreras, Florida State University.

### Immunostaining

Geniculate ganglia were dissected, fixed with 4% paraformaldehyde, washed with PBS, cryoprotected overnight with 30% sucrose, embedded in OCT, and cut at 25 μm. Sections were permeabilied with 1% Triton-X in PBS, treated with 4% normal goat serum in PBS followed by Avidin/Biotin Blocking kit, and immunostained with anti-NeuN (1:1,000, clone A60, biotin-conjugated, MAB377, EMD Millipore) and anti-GFP (1:1,000, GFP-1020, Aves Labs, Inc.). Secondary antibodies were Streptavidin, Alexa Fluor 594 conjugate (1:1,000, S-11227, Life Technologies) and Alexa Fluor 488 goat anti-chicken IgG (H+L) (1:1,000, A-11039, Life Technologies). Sections were mounted with Fluoromount-G (0100-01, Southern Biotech).

## Additional information

**How to cite this article:** Wu, A. *et al*. Breadth of tuning in taste afferent neurons varies with stimulus strength. *Nat. Commun.* 6:8171 doi: 10.1038/ncomms9171 (2015).

## Supplementary Material

Supplementary Information Supplementary figure 1 and Supplementary Methods

Supplementary Movie 1 Responses (dF) of geniculate ganglion cells in vivo in GCaMP3 mice to a panel of 5 tastants presented consecutively in the oral cavity. The movie was collected as one continuous recording but for clarity, has been subdivided into 5 separate episodes. Each episode represents the presentation of one taste stimulus, identified along the bottom. Onset of the stimulus (t=2 sec, duration 5 sec) for each episode is indicated at the top of the left episode. Several neurons respond to the tastants. An example of a neuron that responds best to sucrose (and somewhat to MSG/IMP), S, is identified by a white dot. An example of a neuron that responds to multiple taste stimuli,G, is shown as a magenta dot. Stimuli were 500 mM sucrose, 300 mM MSG (with 1mM IMP), 500 mM NaCl, 50mM citric acid, or 30 ìM cycloheximide + 5 mM quinine.HCl.

## Figures and Tables

**Table 1:** Comparison of EC_50_ values for prototypic sweet, sour, and bitter taste compounds, obtained from whole-nerve recordings (chorda tympani), brief access taste assays, and GCaMP3 recordings.

Taste compound	Chorda tympani whole-nerve recordings	Brief access taste assays	GCaMP3 responses
Sucrose	∼300 mM*	∼120 mM†	137 mM
Citric acid	∼9 mM‡	∼11 mM§	7 mM
Quinine·HCl	∼1 mM||	∼0.2 mM¶	0.2 mM
cycloheximide	∼1 μM||	∼1.1 μM¶	1.7 μM

All data are from C57/BL6 mice which makes these data comparable to the transgenic GCaMP3 mice used in our study (which had been backcrossed to C57/BL6 mice >6 generations). EC_50_ values for chorda tympani recordings and brief access taste assays were estimated from figures in the published reports, whereas those for GCaMP3 responses are from Fig. 4. Comparison data for salt taste are not included because NaCl evokes bimodal behavioural responses (preferred at low concentrations, aversive at high concentrations). Comparison data for umami taste are not included because we did not find exact comparisons in the literature (that is, varying MSG concentrations with a constant 1 mM IMP).

^*^Damak *et al*.^66^

^†^Di Lorenzo and Victor^46^.

^‡^Arai *et al*.^67^

^§^Dotson *et al*.^68^

^||^Yamamoto and Yuyama^15^.

^¶^Boughter *et al*.^69^
