# Supplementary material for: Breadth of tuning in taste afferent neurons varies with stimulus strength
Source: Nat Commun. 2015 Sep 16;6:8171. doi: 10.1038/ncomms9171 (PMC4573454; doi:10.1038/ncomms9171)
Supplement: Supplementary Information — Supplementary figure 1 and Supplementary Methods [file ncomms9171-s1.pdf]

Supplementary material

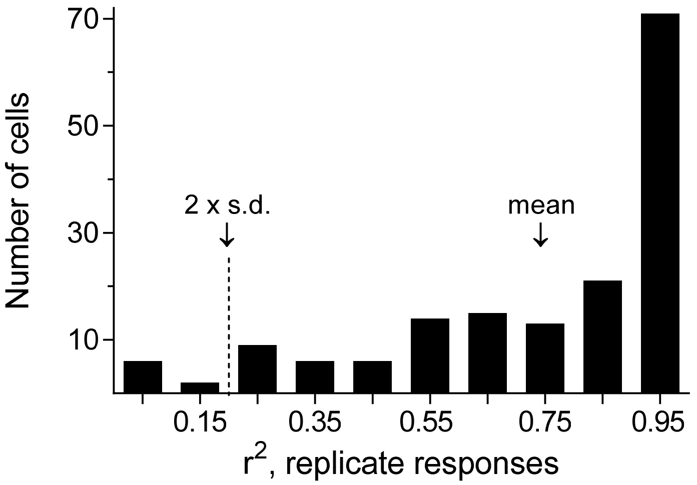

Supplementary Figure 1. Assessing the stability and reliability of tastant-evoked responses (ΔF/F0) recorded from geniculate ganglion cells in GCaMP3 mice. The plot shows the distribution of correlation coefficients across a panel of 5 taste stimuli, presented as in Figure 2D, sucrose, 300 mM; MSG, 100 mM (with 1 mM IMP); NaCl, 250 mM; citric acid, 10 mM; cycloheximide, 1 µM, plus quinine·HCl, 0.3 mM. A value of 1.0 indicates a perfect replication of responses to all 5 stimuli in both presentations. A value of 0 indicates no correlation between the two trials. Dashed lines show mean and 2 x s.d. from the mean. In this sample (n=163), mean – (2 x s.d.) = 0.21

Supplementary Methods, Matlab code for data analysis:

code1: load ROI

```
function [data Iminfo]=load1p(fileno)

[FileName,PathName] = uigetfile('*.tif','Select the TIF file','/Users/');
path=strcat(PathName,FileName);
Iminfo.FileName=FileName;
Iminfo.path=path;

if (nargin == 0)
    INFO=imfinfo(path);
    j=length(INFO);
    x=INFO(1).Width;
    y=INFO(1).Height;
else
    example=imread(path,1);
    [y x]=size(example);
    j=fileno;
end
data=zeros(y,x,j,'int16');

handle=waitbar(0,'Loading image');
for i=1:j
    data(:,:,i)=imread(path,i);
    waitbar(i/j,handle)
end
close(handle);
disp(path);

return
```

code2: analyze ROI

```
% This scripts is to analyze data in the case that too many roi in single
% image.  The first step is to draw roi in ImageJ and then save as zip file.
% As the oval roi in ImageJ doesn't have angle,so to draw ellipse in
% matlab, the Angle is default as zero.
%

%% load image
clc;clear;close all;
[Im,Iminfo]=load1p;

Iminfo.frameNo=size(Im,3);
ref=mean(Im,3);

%% load roi
Iminfo.roipa=[Iminfo.path(1:end-3) 'zip'];
```

```
Roi=ReadImageJROI(Iminfo.roipa);
Roi_num=length(Roi);
[row,col]=size(ref);
mRoi=cell(1,Roi_num);
%% draw roi in matlab matrix
for i=1:Roi_num
   if strcmpi(Roi{1,i}.strType,'Oval')  %check the Roi type
       % get the center position
       xc=1/2*(Roi{1,i}.vnRectBounds(2)+Roi{1,i}.vnRectBounds(4));
       yc=1/2*(Roi{1,i}.vnRectBounds(1)+Roi{1,i}.vnRectBounds(3));
       a=1/2*(Roi{1,i}.vnRectBounds(4)-Roi{1,i}.vnRectBounds(2));
       b=1/2*(Roi{1,i}.vnRectBounds(3)-Roi{1,i}.vnRectBounds(1));
       alfa=linspace(0,360,16).*(pi/180);  % use 16 points to draw the ellipse
       % get points on the ellipse
       xi=xc+a.*cos(alfa);
       yi=yc+b.*sin(alfa);
       % save the parameter of the roi
       % xi yi          the points on the ellipse
       % center         the position of the ceter of the ellipse
       % area           the area of the ellipse
       % x              the row range of the image
       % y              the column range of the image
       % BW             mask of the roi
       %ncEllipBounds    the bounds of the ellipse [ top left bottom right]
       roi.xi=xi;
       roi.yi=yi;
       roi.center=[xc yc];
       roi.area=polyarea(roi.xi,roi.yi);
       roi.x=[1 row];
       roi.y=[1 col];
       roi.BW=poly2mask(xi,yi,row,col);
       roi.ncEllipBounds=Roi{1,i}.vnRectBounds;

   else
     if strcmpi(Roi{1,i}.strType,'Polygon') % check the roi type
        % xi yi            the points on the  polygon
        % x y              the row/column range of the image
        % BW               the mask of the roi

        % get the points and center on the polygon
        xi=[Roi{1,i}.mnCoordinates(:,1); Roi{1,i}.mnCoordinates(1,1)];
        yi=[Roi{1,i}.mnCoordinates(:,2); Roi{1,i}.mnCoordinates(1,2)];
        x=[1 row];
        y=[1 col];
        xc=mean(xi(1:end-1));
        yc=mean(yi(1:end-1));
        roi.xi=xi;
        roi.yi=yi;
        roi.center=[xc yc]
        roi.BW=poly2mask(xi,yi,row,col);
     else
```

```
         error('roi type must be oval or polygon')
      end


    end
    mRoi{i}=roi;
end
% plot the roi
figure(1); imagesc(ref);axis image;axis off;
for i=1:Roi_num
        hold on;
        plot(mRoi{1,i}.xi,mRoi{i}.yi,'Color','k','LineWidth',1);
        text(mRoi{i}.center(1), mRoi{i}.center(2), num2str(i),...
            'Color', 'k', 'FontWeight','Bold');
end

%% calculate the intensity dynamics in roi regions
for i=1:Roi_num
    A=repmat(mRoi{1,i}.BW,[1,1,size(Im,3)]); A=int16(A);
    df1=squeeze(sum(sum(Im.*A,1),2))/sum(sum(mRoi{1,i}.BW,1),2);
    df1=df1';
    dF(i,:)=df1;
end

 %% plot dF vs time
 F0=repmat(mean(dF(:,1:50),2),1,size(dF,2));
 dF_F=(dF-F0)./F0;
 N=size(dF_F,1);
 for i=1:N
     df_f2=smooth(dF_F(i,:),3);
     df_f(i,:)=df_f2;
 end
 Iminfo.df_f=df_f;
 Iminfo.df=dF;
 Iminfo.F0=F0;

 figure;plot(1:size(df_f,2),df_f);
 hold on; ylabel('dF/F');xlabel('Frame No.');
 hold off;
 figure;
 for i=1:20
     if i<=size(df_f,1)
         subplot(5,4,i);
         plot(1:size(df_f,2),df_f(i,:));
         ylabel('dF/F');xlabel('Frame No.');
         title(['cell' ' ' num2str(i)]);
     end
 end
 if size(df_f,1)>20;
     figure;
     for i=21:size(df_f,1);
```

```
        subplot(5,4,i-20);
        plot(1:size(df_f,2),df_f(i,:));
        ylabel('dF/F');xlabel('Frame No');
        title(['cell' ' ' num2str(i)]);
    end
end

colv=colova;
figure;imagesc(df_f); colormap(colv);
colorbar;
xlabel('Frame No.');
ylabel('Number of cells');
title('Geniculate Ganglion neuron response profile');
%% Get peak value in dF_F


Peakk = df_f(:,50:110);
 for n = 1:N
   Peak(n) = max(squeeze(Peakk(n,:)));
 end
nn = 1:N;

 %% Get duration in dF_F
s=std(dF_F(:,1:50),0,2);
[r c]=find(dF_F(:,50:100)>3*repmat(s,1,size(dF_F(:,50:100),2)));  % respone limited to frame 50
to end;
for i=1:size(dF_F,1)
    fram_resp=c((find(r==i)));
    if isempty(fram_resp)
        ind=num2str(i); disp([' Cell ' ind  ' does not response to stimulus']);
        dur(i)=0; Peak(i)=0;
        Start_fram(i)=0;End_fram(i)=0;
    else
        ind=num2str(i);
        dur(i)=max(fram_resp)-min(fram_resp)+1;
        Start_fram(i)=50+min(fram_resp)-1;
        End_fram(i)=50+max(fram_resp)-1;


    end
end


Peak=Peak';
Iminfo.Peak=Peak;


[filename pathname]=uiputfile('.mat','Save the Results','/Users/');
save(fullfile(pathname,filename),'Iminfo');
```

code3: get peak value

```
% This script is used to load peak value of different trials in a batch;
% all mat file containing Iminfo.Peak of different trial must be saved in
% one fold. No other mat files are permittted in this fold.
clear;close all;clc;
path=uigetdir('/Users/');
B= dir(fullfile(path,'*.mat'));
B = struct2cell(B);
num = size(B);

for k =0:num(2)-1
    z(k+1) = B(size(B,1)*k+1);
end
df_f=[];
for k = 1:num(2)
    newpath = strcat(path,'/',z(k));
    load((char(newpath)),'Iminfo');
    df_fp(:,k)=Iminfo.Peak;
end
colv=colova;
df_fp=df_fp';
figure;imagesc(df_fp);colormap(colv);
ylabel('Trial Number'); xlabel('Cell Number');
title('Peak value in different trial');
df_fpavg=mean(df_fp,1);
[filename pathname]=uiputfile('.mat','Save the Results','/Users/');
save(fullfile(pathname,filename),'df_fp','df_fpavg');
```

code4: calculate h value & dendrogram

```
clear;close all;clc;
 %% Load the Excel File
 clear;
 [FileName PathName]=uigetfile('*.xls','Select the Excle file','/Users/');
 PathData = strcat(PathName,FileName);
 [peak_nor text alldata]=xlsread(PathData);

 %% Get H value
 % normalize all peak value of different cell to the 5th stimulus;
 % calculate the H value of entropy equation;
 % H(P1,P2,...Pn)=-K*ΣPi*logPi;Pi is the peak df/f of single neuron to the
 % ith stimulus, K is a constant determined by number of stimulus. K=1/log10(n); n is the
number of stimulus;
 N=4;  % N is the number of stimulus;
 K=1/log10(N);        % calculate the constant K
 peak_total=sum(peak_nor,2);  % get the total response of single neuron to all stimulus
 p=peak_nor./repmat(peak_total,1,N); % calculate proportional response of single neuron to
each stimulus
 p_logp=p.*log10(p);
 p_logp(isnan(p_logp))=0;  % When p is zero, the log10(p) would not be a number, in this case
```

```
log10(p) is asigned to be zero
 H=-K*(sum(p_logp,2));      % calculate H value of each cell according to the function
H(P1,P2,...Pn)=-K*ΣPi*logPi;

 figure;
 plot(H','b*-');
 xlabel(' Cell Index'); ylabel('H Value');
 title('H Value of Different Cell');
figure;hist(H);
set(gca,'XLim',[0 1],'XTick',0:0.1:1);
xlabel(' Value (H) '); ylabel('Number of Cell');
cell_No=num2str(length(H));t=[' Entropy ' 'Distribution ' '(N=' cell_No  ')'];
title(t);

 %% Using Pearson's r and average-linking method to get dendrogram cluster
 D=pdist(peak_nor,'correlation'); % get the Pearson's r and p value;
 linkage_r=linkage(D,'average');
 [c M]=cophenet(linkage_r,D);
 r=corr(D',M','type','spearman');
 figure;
 lin_rh=dendrogram(linkage_r,0,'orientation','right');
 xlabel('Dendrogram Cluster Distance');ylabel('Cell Index');
 title(' Pearson_s R and Average-linking Clustering');
 %% Using Euclidean distance and average-linking method to get drendrogram cluster
 D=pdist(peak_nor,'euclidean'); % get the euclidean distance
 linkage_eucli=linkage(D,'average');
 [c M]=cophenet(linkage_eucli,D);
 r2=corr(D',M','type','spearman');
 figure;lin_euclih=dendrogram(linkage_eucli,0,'orientation','right');
 xlabel('Dendrogram Cluster Distance');ylabel('Cell Index');
 title(' Euclidean Distance and Average-linking Clustering');
 save([PathName 'H_r_dist_value'],'H','alldata','peak_nor','r','r2');
```
