# Supplementary figures and images for: Breadth of tuning in taste afferent neurons varies with stimulus strength
Source: Nat Commun. 2015 Sep 16;6:8171. doi: 10.1038/ncomms9171 (PMC4573454; doi:10.1038/ncomms9171)

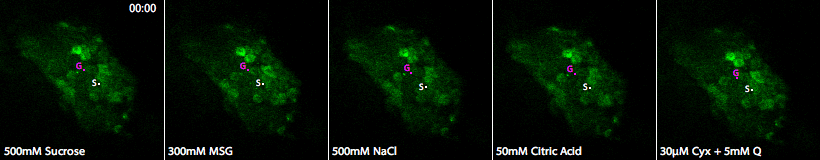

Supplement: Supplementary Movie 1 — Responses (dF) of geniculate ganglion cells in vivo in GCaMP3 mice to a panel of 5 tastants presented consecutively in the oral cavity. The movie was collected as one continuous recording but for clarity, has been subdivided into 5 separate episodes. Each episode represents the presentation of one taste stimulus, identified along the bottom. Onset of the stimulus (t=2 sec, duration 5 sec) for each episode is indicated at the top of the left episode. Several neurons respond to the tastants. An example of a neuron that responds best to sucrose (and somewhat to MSG/IMP), S, is identified by a white dot. An example of a neuron that responds to multiple taste stimuli,G, is shown as a magenta dot. Stimuli were 500 mM sucrose, 300 mM MSG (with 1mM IMP), 500 mM NaCl, 50mM citric acid, or 30 ìM cycloheximide + 5 mM quinine.HCl. [file ncomms9171-s2.gif]
